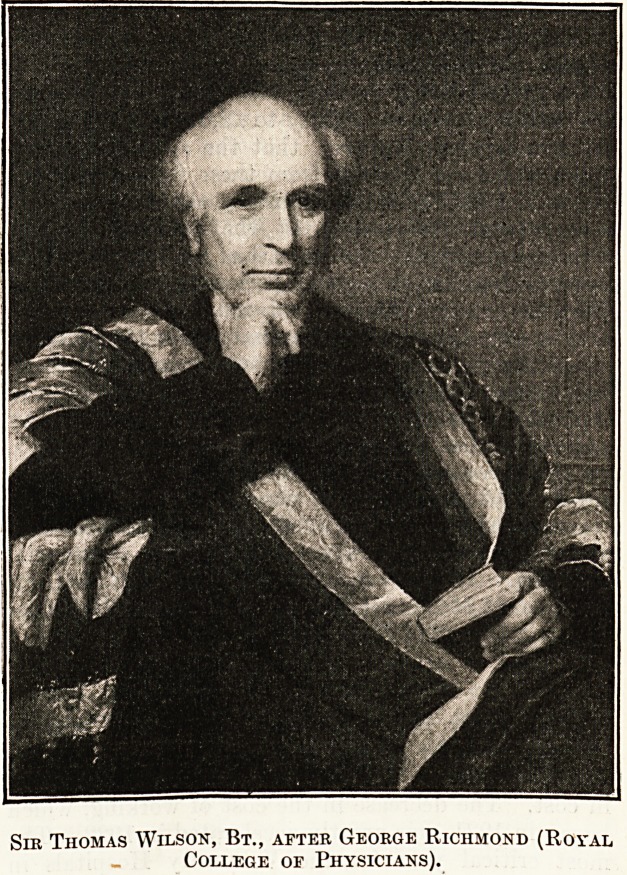# Middlesex Hospital School

**Published:** 1923-11

**Authors:** 


### MIDDLESEX HOSPITAL SCHOOL.

### PORTRAITS OF FOUNDERS.

THE Medical School of the Middlesex Hospital was founded by Francis Hawkins, Thomas Watson, Charles Bell, Herbert Mayo, James Moncrieff Arnott and Edward W. Tuson. Mr. A. E. Webb-Johnson, C.B.E., D.S.O., the Dean of the School, has been anxious to obtain a complete collection of the portraits in oils of the founders of the School to be hung in the Dean's room, but only one of them, that of Francis Hawkins, which was presented by his old pupils, had hitherto been acquired, while no picture of Tuson appears to be in existence. The portrait of Herbert Mayo, by Lonsdale, is still in the possession of a representative of the family, and there is reason to believe that some day it will hang upon the walls of the school "where," says Mr. Webb-Johnson, "his name will always be held in honour." The school is, however, already the fortunate possessor of the beautiful engraving by David Lucas of Lonsdale's portrait of Mayo, presented by the late Sir James Galloway.

### A Generous Gift.

Writing recently to the Earl of Athlone, the Chairman of the Hospital, Mr. Webb-Johnson, after reminding his correspondent of these facts, continued: "With great patience and skill and with remarkable success Mr. Dorofield Hardy has made copies for me of the portraits of the other three founders of the school—that of Sir Thomas Watson from the well-known picture by George Richmond in the Royal College of Physicians, that of Sir Charles Bell from the portrait by Stevens in the National Portrait Gallery, and that of James Moncrieff Arnott from the portrait in the Royal College of Surgeons, probably by Lane, a pupil of Lawrence. I ask your acceptance of these three pictures for the medical school in order to make the collection of the portraits of the founders as complete as possible. I cherish the hope that, if hung in the Dean's Room, they will be a constant reminder to successive holders of that office of the greatness of the inheritance of which they are the trustees for the time, for in the continual remembrance of a glorious past individuals and Institutions, as well as nations, find their noblest inspirations." The portraits have now been hung in the Dean's room, where they are open to the inspection of visitors, and we are happy to be able to reproduce photographs of them specially taken for the Hospital and Health Review by the courtesy of the Dean. We append a few particulars of the careers of these three of the six original founders who in 1835 signed a petition from the hospital staff to the Governors for the establishment of a medical school.

Sir Charles Bell, after Stevens (National Gallery).

### Sir Charles Bell.

Sir Charles Bell (1774-1842) was born at Edinburgh, and combined artistic pursuits with his medical studies. At the age of thirty he came to London, where he chiefly devoted his time to investigations on the nervous system. In 1830 he published "The Nervous System of the Human Body," which embodied the completed result of his experiments. These are stated on good authority to be the greatest discoveries in physiology since Harvey's on the circulation of the blood. Bell's reputation as a surgeon may be illustrated by the fact that he was sent to Haslar Hospital in 1809 to attend the wounded from Coruña and to Brussels in 1815 after Waterloo. He was connected with the Middlesex Hospital from 1812 to 1836, when he returned to Edinburgh to occupy the Chair of Surgery.

### Sir Thomas Watson.

Sir Thomas Watson (1792-1882), first baronet, was educated at Bury St. Edmunds Grammar School and Cambridge, where he graduated as tenth Wrangler and became a Fellow of his college. He also studied under Abernethy at St. Bartholomew's, and spent one session at Edinburgh. He was Physician to the Middlesex Hospital from 1827 to 1843, and also held professorships at King's College. In 1843 he published "Lectures on the Principles and Practice of Physic," which for thirty years maintained its place as the chief English text-book on the subject. He was successively Physician Extraordinary and Physician in Ordinary to the Queen, and attended the Prince Consort in his last illness. He was five times re-elected President of the Royal College of Physicians.

### James Moncrieff Arnott.

James Moncrieff Arnott, Professor of Surgery at King's College, was Surgeon to the Middlesex Hospital from 1831 to 1848.

James Moncrieff Arnott, probably after Lane (Royal College of Surgeons).

The Other Three.

Francis Hawkins (1794-1877) was educated at Merchant Taylors School and Oxford, where he gained the Newdigate Prize and became a Fellow of his college. He was appointed physician to the Middlesex Hospital in 1824, and was connected with it for thirty-four years. He also held the first Professorship of Medicine at King's College, and was Physician to the Royal Household under William IV. and Victoria. Herbert Mayo (1796-1892) made a great reputation as a physiologist and anatomist. He was a pupil of Sir Charles Bell at the Middlesex Hospital. Besides his appointments at that Hospital, he also held professorships at King's College, which he resigned in 1836. He was the author of manv treatises, including one on Mesmerism and Superstitions. Edward W. Tuson was one of the first lecturers on anatomy and surgery in the new school, and was largely concerned in the establishment of the museum.

### NEED OF A HEALTH MINISTRY IN IRELAND.

The Annual Council Meeting of the Irish Nurses' and Midwives' Union was held recently in Dublin. Miss Annie Smithson moved: "That the Council should consider how the Irish Nurses' and Midwives' Union could press the Government to set up immediately a Ministry of Health." She said that Public Health matters need looking after very badly in Ireland, and particularly Child Welfare schemes should be extended.

Dr. Rowlette, speaking in support of the motion, said that there could be no prosperity in Ireland without good health. He was not a politician, and had no very definite ideas as to the best method to pursue to establish a Ministry of Health. The Irish Medical Committee had already pressed the matter on the Government by means of resolutions, as he understood the nurses had done, and he did not think it was much use individual bodies sending resolutions any more. He suggested that all bodies or persons interested should unite in pressing the matter. The Nurses' Union was in a strong position, as they had expert knowledge of the needs of the people, and, secondly, they were in touch with Labour, and therefore might act as a link between the other bodies interested and the Dail through the Labour Party, the only party which had made Health part of their programme. The nurses' opinion should be given as from those who had knowledge of the needs of the community. Efficiency was essential, and, therefore, conditions of service must be satisfactory. Ireland was very backward, and far behind other civilized countries in health matters. In tuberculosis the improvement was far less than in other countries, and the infant mortality rate was terribly high. In Dublin the infant mortality rate was more than 50 per cent. higher than in London. Even at present the Act under which medical inspection of school children could be carried out had been on the Statute book for four years, and only now were some few local authorities thinking of putting it into force.

Sir Thomas Wilson, Bt., after George Richmond (Royal College of Physicians).

## Figures and Tables

**Figure f1:**
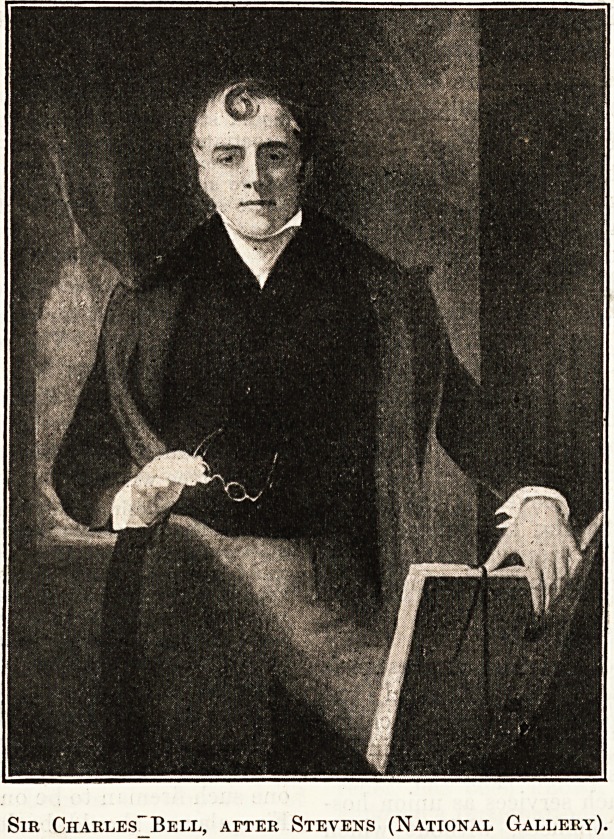


**Figure f2:**
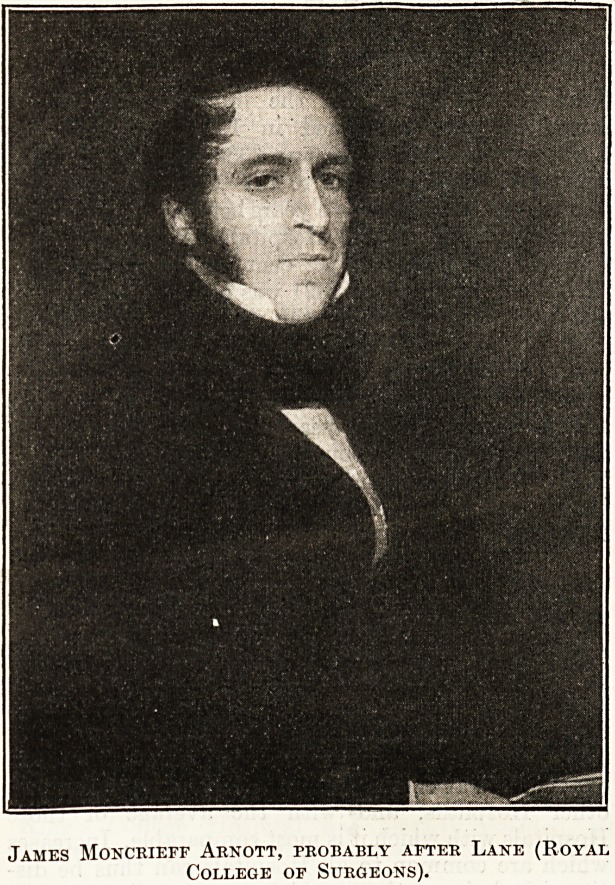


**Figure f3:**